# Impact of a controlling coaching style on athletes’ fear of failure: Chain mediating effects of basic psychological needs and sport commitment

**DOI:** 10.3389/fpsyg.2023.1106916

**Published:** 2023-02-03

**Authors:** Qing Hu, Peng Li, Bo Jiang, Bo Liu

**Affiliations:** ^1^School of Educational Science, Jiangsu Second Normal University, Nanjing, China; ^2^School of Physical Education, Jiangsu Second Normal University, Nanjing, China; ^3^Huaqiao Jishan Middle School, Suzhou, China

**Keywords:** controlling coaching style, basic psychological need, fear of failure, sports commitment, enthusiastic commitment, constrained commitment, chain mediating effects, athletes

## Abstract

Controlling coaching style is a key predictor of athletes’ fear of failure, but the mediating mechanisms underlying this relationship require further exploration. Based on the self-determination theory (SDT) and the hierarchical model of intrinsic and extrinsic motivation (HMIEM), this study investigated the effect of a controlling coaching style on athletes’ fear of failure, as well as the chain mediating effects of basic psychological needs and sport commitment. A questionnaire survey of 252 active athletes was administrated using scales for fear of failure, controlling coaching style, sport commitment, and basic psychological needs. The results indicated that a controlling coaching style was negatively correlated with basic psychological needs and indirectly affected athletes’ fear of failure *via* basic psychological needs and constrained commitment. The chain mediating effect of basic psychological needs on enthusiastic commitment was not significant, but it was for constrained commitment. In conclusion, the impact of a controlling coaching style on athletes’ fear of failure *via* basic psychological needs is manifested through the strengthening of constrained commitment rather than the weakening of enthusiastic commitment. These findings contribute to a deeper understanding of why and how a controlling coaching style influences athletes’ fear of failure. Coaches should seek more scientific and effective ways to instruct their athletes.

## 1. Introduction

High-performance sports are extremely competitive, and performance failure is the most prominent situational stress for athletes (Smith et al., 1990). Many elite athletes experience fear of failure (FF) when participating in such sports (Correia and Rosado, 2018), which is a negative emotion experienced when an individual engages in achievement-oriented activities and predicts they will not meet certain set goals (Conroy et al., 2001). Although FF can motivate athletes to remain competitive and strive for good results to some extent (Martin and Marsh, 2003), numerous studies have highlighted its negative impact, such as triggering athletes’ withdrawal (Sagar et al., 2007), performance decline (Sagar et al., 2009), anti-social sports behavior (Sagar et al., 2011), burnout (Gustafsson et al., 2017), and somatic and cognitive anxieties (De Muynck et al., 2020). Therefore, the critical factors affecting athletes’ FF and the related mechanism must be examined to provide effective scientific methods for coping with FF and improving their competitive mental health.

Vallerand et al. (1997) proposed the hierarchical model of intrinsic and extrinsic motivation (HMIEM), which includes three levels of motivational factors: global, contextual, and situational. At the situational level, factors affect situational motivation through three basic psychological needs (BPN), affecting situational consequences, which refer to the cognitive, emotional, or behavioral consequences for a specific task at a specific time. The self-determination theory (SDT) posits that the psychological environment created by significant others (such as coaches) and its resultant impact on the motivational process of athletes are crucial in determining the quality and consequences of sports participation (Deci and Ryan, 2000). Hence, this study aimed to examine how the situational factor of coaching style affects the emotional consequences of athletes’ participation in high-performance sports through BPN and situational motivation. Hence, this study aimed to examine how the situational factor of coaching style (e.g., CCS) affects the emotional consequences (e.g., FF) of athletes’ participation in high-performance sports through BPN and situational motivation (e.g., EC and CC).

The coach is a vital interpersonal factor affecting athletes (Horn, 2002; Amorose and Anderson-Butcher, 2007), and the management of interpersonal relationships between coaches and athletes is crucial to shaping the latter’s psychological experiences (Bartholomew et al., 2010). There are two types of coaches based on coaching style: autonomous and controlling. Coaches with an autonomous style often allow athletes to participate in decision-making and acknowledge and respect their views and feelings. Coaches with a controlling style often employ coercive, threatening, and authoritarian methods to impose their ideas on athletes while ignoring or dismissing the latter’s perspectives and emotions (Hodge and Lonsdale, 2011). In a study about physical education, Bartholomew et al. (2018) found that a controlling coaching style (CCS) was associated with adolescent students’ FF. Moreover, González-Hernández et al. (2019) revealed a significant positive relationship between a CCS and athletes’ FF for high-performance sports. A high CCS level was found to mediate a moderate level of FF in athletes.

Chinese coaches differ from their Western counterparts because of the particularity of China’s training system and model for high-performance sports. They often have dual identities of coaches and pseudo-parents. Coaches demonstrate paternalistic benevolence and dignity when mandating that athletes obey and respect various behavioral boundaries expected in the sporting environment (Si et al., 2011). When the coach–athlete relationship is poorly managed, athletes may perform poorly during competitions and their personal growth and mental health may be adversely affected. Hence, it is critical to examine the impact of a CCS on athletes for the Chinese culture and system. The findings can help advance the understanding of coaching styles’ impact on athletes with different cultural and institutional backgrounds.

The BPN theory, a sub-theory under the SDT framework, proposes that the social environment optimizes the internal functions of the human body by satisfying the three BPN of autonomy, competence, and relatedness, leading to improved performance levels (Deci and Ryan, 2014). Need for autonomy represents the need to feel control in decision-making process regarding own choices and activities (Reinboth and Duda, 2006). Need for competence refers to need to perceive one’s behavior and interaction with others and the world, as successful; to feel competent in different situational contexts, and confident in own abilities. The need for relatedness represents need to connect with others, to be accepted, and to achieve reciprocal interpersonal relationships (Reinboth and Duda, 2006). When the environment hinders the satisfaction of these BPN, individuals’ autonomous motivation, job performance, and well-being reduce (Ryan and Deci, 2000). This indicates that coaching styles’ impact on athletes’ FF could be understood through BPN. Sports studies based on the BPN theory primarily examine the positive impacts of the autonomous coaching style on BPN satisfaction, encompassing adolescent to adult athletes and yielding relatively consistent results (e.g., Gagne et al., 2003; Mageau and Vallerand, 2003; Amorose and Anderson-Butcher, 2007; Adie et al., 2008; Balaguer et al., 2008; Alvarez et al., 2009). By comparison, there is a lack of studies on the relationship between a CCS and the satisfaction of BPN, and those arriving at inconsistent conclusions.

Some studies concluded that a CCS negatively correlated with BPN satisfaction (Curran et al., 2014; Yang et al., 2020). However, Blanchard et al. (2009) found that a coach’s controlling interpersonal style negatively correlated with autonomous needs satisfaction but was not significantly related to the other two BPN. According to Bartholomew et al. (2011) and Pulido et al. (2018), there is no correlation between a controlling style and BPN satisfaction. This may be because most previous studies on BPN are based on the overall rather than specific interpersonal context between coaches and athletes. In these studies, the commonly used expressions included “I feel close to other people” from the BPN satisfaction scale (BNSSS) (Ng et al., 2011) and “I feel that I am part of the team” from the needs satisfaction scale (Blanchard et al., 2009).

The factors affecting BPN are diverse. For athletes, the three most important factors are the coach, teammates, and parents (Keegan et al., 2009, 2010), with each factor playing a different role (Chu and Zhang, 2019). When this kind of questions are raised for athletes, their coach is not the only factor they consider, the impacts of their teammates and family members are also included. Consequently, the negative impact of a CCS on the satisfaction of athletes’ BPN might be underestimated. Some researchers made modifications to address this problem. For example, Raabe and Zakrajsek, (2017) modified the BNPS scale (Deci et al., 1989) to assess the impact of a CCS on the satisfaction of athletes’ three BPN based on the specific interpersonal context of coaches and teammates, and discovered a significant influence by their coach and teammates. However, they did not measure the impact of different coaching styles on BPN satisfaction. Hence, this is the first study to examine the relationship between a CCS and BPN satisfaction based on the specific interpersonal context of coaches and athletes. Previous studies have shown that BPN satisfaction negatively predicted FF in athletes (Conroy et al., 2007). This led to the proposal of Hypothesis 1: A negative correlation exists between a CCS and athletes’ BPN, and the latter mediates CCS and FF.

The motivational force of athletes is influenced by factors other than the psychological environment created by significant others (such as the coach) (Deci and Ryan, 2000). Sport commitment (SC) is a motivating force that links athletes to their sports and affects the persistence and functioning of their sports behavior (Scanlan et al., 2003). The SC serves as a factor protecting athletes’ health against destructive reactions such as FF (Bélanger et al., 2013; González-Hernández and Muñoz-Villena, 2019); however, it also prompts them to pursue tremendous goals, set unrealistic expectations, or identify excessively with the meaning of sport (Olusoga and Kentta, 2017). This leads to them perceiving a lack of achievement on their part, which is a cause for concern (Madigan et al., 2016; González-Hernández and Muñoz-Villena, 2019), suggesting it may not suffice to examine the role of SC from a single perspective.

Scanlan et al. (2016) proposed a two-dimensional SC model (SCM) to replace the earlier unidimensional perspective of SC. The SCM divides SC into enthusiastic commitment (EC) and constrained commitment (CC). EC (i.e., “want”) represents an individual’s desire for and dedication to a sport, while CC (i.e., “have to”) reflects an individual’s obligation to and passive responsibility for a sport. Motivation can be autonomous or controlled in SDT (Ryan and Deci, 2017), which shares conceptual similarities with the two dimensions of commitment (Scanlan et al., 2013). However, autonomous and controlled motivations are considered distal psychological variables affecting motivated behaviors in the background, whereas SC is a psychological state and proximal psychological variable at the situational level. It connects athletes to a specific commitment goal, such as a club, team, or sports activity, and has a direct and immediate effect on behavior (Boiché and Sarrazin, 2009). Considering this, the SCM was applied in this study to examine the impact of a CCS on athletes’ FF.

Several studies support the positive predictive effect of social constraints on athletes’ CC (Young and Medic, 2011; Santi et al., 2014; Scanlan et al., 2016; O’Neil and Hodge, 2020), but conclusions on the impact of social constraints on athletes’ EC and CC are inconsistent. Some studies have shown that social constraints have no effect (Sousa et al., 2007; Scanlan et al., 2016; O’Neil and Hodge, 2020) or a negative impact (Carpenter et al., 1993; Carpenter and Scanlan, 1998; Santi et al., 2014). This led to the proposal of Hypothesis 2: The relationship between CCS and FF is mediated by CC. No assumption was made on the relationship between CCS and athletes’ EC because it has not been clarified before.

**Figure 1:**
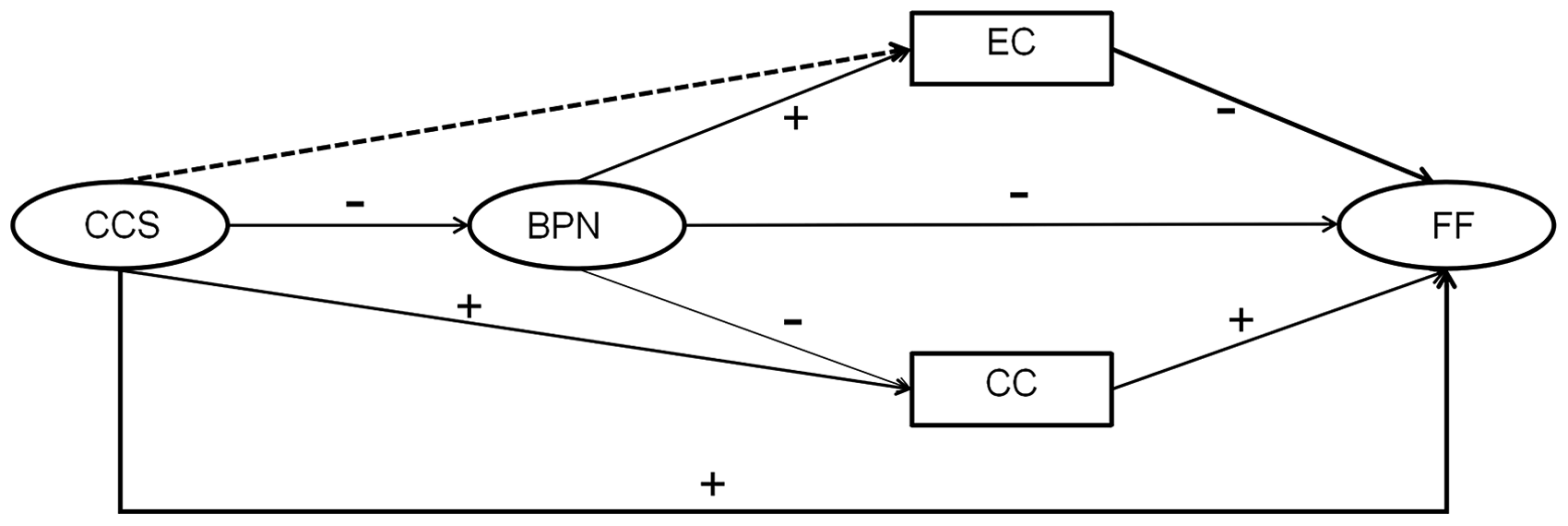
Hypothetical model of the relationship between CCS and FF. CCS, controlling coaching style; BPN, basic psychological need; CC, constrained commitment; EC, enthusiastic commitment; FF, fear of failure. The positive sign between CCS and FF indicates that CCS positively affects FF, thus higher levels of CCS are associated with more FF. The minus sign indicates between CCS and BPN indicates that CCS negatively affects BPN, thus higher levels of CCS are associated with less BPN. The minus sign indicates between BPN and FF indicates that BPN negatively affects FF, thus higher levels of BPN are associated with less FF. a CCS can predict athletes’ FF through BPN. The more CC were perceived to be, the lower athletes’ BPN, leading to a stronger FF. The positive sign between CCS and CC indicates that CCS positively affects CC, thus higher levels of CCS are associated with more CC. The positive sign indicates between CC and FF indicates that CC positively affects FF, thus higher levels of CC are associated with more FF and CCS can predict athletes’ FF through CC. The more CCS were perceived to be, the stronger the CC, leading to a stronger FF. The dotted line between CCS and EC indicates that no assumption was made on the relationship between CCS and athletes’ EC, because it has not been clarified before. The minus sign indicates between EC and FF indicates that EC negatively affects FF, thus higher levels of EC are associated with less FF. The positive sign between BPN and EC indicates that BPN positively affects EC, thus higher levels of BPN are associated with more EC. The minus sign indicates between BPN and CC indicates that BPN negatively affects CC, thus higher levels of BPN are associated with less CC. BPN had a chain mediating effect on CC, mediated by a CCS and athletes’ FF, The more controlling their coaches were perceived to be, the lower athletes’ BPN and the stronger the CC, leading to a stronger FF. The chain mediating effect of BPN and EC has not yet been hypothesized, because it has not been clarified before.

To date, several studies have simultaneously tested the full sequence of motivations described in the HMIEM by Vallerand et al. (1997). These studies investigated how social factors predicted BPN, which predicted self-determination and motivation, resulting in a variety of outcomes (Hollembeak and Amorose, 2005; Blanchard et al., 2009). A positive correlation between BPN and unidimensional SC was also supported (Pulido et al., 2018). However, few studies have incorporated the SCM into the hierarchical model for consideration. It is therefore important to examine the impact of the various commitment types of individuals on the contextual motivation sequence of social factors. Studies have shown that the three BPN are positively and negatively correlated with EC and CC, respectively (Zhang and Yu, 2021). In the current study, Vallerand’s HMIEM and the SCM were combined to investigate how a CCS affected athletes’ FF through BPN and the multidimensional perspective of EC and CC. This led to the proposal of Hypothesis 3: BPN and CC have a significant chain mediating effect. The chain mediating effect of BPN and EC has not yet been hypothesized. The overall hypothetical model is shown in Figure 1.

## 2. Materials and methods

### 2.1. Participants’ recruitment

In order to investigate the influence of CCS on athletes’ FF accurately, we invited athletes who served in a professional sports team, obtained the national certificate, and frequently participated in the national level or above competitions as our subjects, while amateur sports enthusiasts and physical educational students in the school are not our survey subjects.

The cluster sampling method was used to select 300 active athletes from sports teams in six provinces in China (Table 1). The survey process was divided into a pre-survey and a formal survey. The questionnaire was reworked to address the pre-survey’s vague expressions and linguistic ambiguities. Offline distribution of Paper-and-pencil questionnaire survey was used in the survey process. Prior to data collection, participants and their coaches/parents were informed on the purpose, procedures, risks, and benefits of the study, and consent was obtained from sports team administrators, parents of minor athletes, coaches, and athletes. Accordingly, 300 paper questionnaires were distributed. During the administration process, athletes completed the questionnaire in a separate room without the participation of sports team managers and coaches, ensuring participant anonymity and confidentiality throughout the process. In addition, participation was voluntary, and participants provided their written informed consent to participate in the study. After eliminating questionnaires with frivolous responses (answered with irregularity), 252 valid ones were retrieved, representing an effective recovery rate of 84%, 121 men (48%) and 131 female (52%). Participants were aged in their 10–14 years old (*n* = 32, 12.7%), 15–19 years old (*n* = 114, 45.2%), 20–24 years old (*n* = 68, 27.0%), and 25 and above (*n* = 38, 15.1%). There were 120 athletes in the group event, and 132 athletes in the individual events. There were 67 elite level athletes (26.6%), 88 National Level 1 athletes (34.9%), and 97 National Level 2(38.5%).

**Table 1:** Demographic characteristics of the participants (*n* = 252).

Variable	Number	Percentage (%)
Sports events		
Group events (*n* = 120)		
Handball	15	6.0
Rugby	16	6.3
Basketball	26	10.3
Football	18	7.1
Rhythmic gymnastics	12	4.8
Synchronized swimming	12	4.8
Rowing	21	8.3
Individual events (*n* = 132)		
Badminton	43	17.1
Gymnastics	26	10.3
Martial arts	40	15.9
Athletics	23	9.1
Gender
Male	121	48.0
Female	131	52.0
Age (years)
10–14	32	12.7
15–19	114	45.2
20–24	68	27.0
≥25	38	15.1
Sports level
Elite athlete	67	26.6
Level 1	88	34.9
Level 2	97	38.5

This is an appropriate sample size for avoiding inaccurate estimates of standard errors and fit indexes, according to the criterion proposed by Jackson (2003) for structural equation modeling (SEM). This author suggested that an ideal sample size should meet the ratio of 20 cases per each parameter to be estimated in the model or a less ideal ratio of 10 cases per parameter. Considering that our larger model contained 17 parameters to be estimated, the range of the ideal sample size would be between 170 and 340 participants.

### 2.2. Research tools

#### 2.2.1. Controlling coaching style

The 15-item Controlling Coach Behaviors Scale (CCBS; Bartholomew et al., 2010) was employed to assess athletes’ perceptions of controlling behaviors and strategies conveyed by their coach. It contains four subscales measuring: controlling use of rewards (four items; e.g., “My coach tries to motivate me by promising to reward me if I do well”), negative conditional regard (four items; e.g., “My coach is less friendly with me if I do not make the effort to see things his/her way”), intimidation (four items; e.g., “My coach shouts at me in front of others to make me do certain things”), and excessive personal control (three items; e.g., “My coach expects my whole life to center on my sport participation”). Participants provided answers using a seven-point Likert scale (1 = Strongly disagree, 7 = Strongly agree), the higher the score, the more rigid the coach’s control. The Cronbach’s α coefficient of the scale was 0.93. In this study, the Cronbach’s α coefficient of the scale was 0.913.

#### 2.2.2. Sport commitment

The Sport Commitment Questionnaire-2 (SCQ-2; Scanlan et al., 2016) was employed to measure athletes’ sport commitment (SC) to their current sport participation. The 11-item SCQ-2 contains two subscales measuring the two dimensions of sport commitment proposed in the SCM (Scanlan et al., 2013, 2016): enthusiastic commitment (EC; six items; e.g., “I am willing to do almost anything to keep playing this sport”), and constrained commitment (CC; five items; e.g., “I feel I have to keep playing this sport, even though I do not want to”). Participants responded to each item using a five-point Likert scale (1 = Strongly disagree, 5 = Strongly agree), the higher the score, the stronger the athlete’s SC. The composite reliabilities of the EC and CC subscales were 0.92 and 0.78, respectively. In this study, the Cronbach’s α coefficients of the EC and CC subscales were 0.897 and 0.850, respectively.

#### 2.2.3. Basic psychological need in relationships

The Basic Psychological Needs Scale was developed by La Guardia et al. (2000). This questionnaire was designed for use with respect to basic psychological need (BPN) satisfaction in particular relationships. The 9-item scale contains three subscales: Autonomy (three items; e.g., “When I am with XXXXXXX, I feel free to be who I am”), Competence (three items; e.g., “When I am with XXXXXXX, I feel like a competent person”), and Relatedness (three items; e.g., “When I am with XXXXXXX, I feel loved and cared about”), participants provided answers using a seven-point Likert scale (1 = not at all true, 4 = somewhat true, 7 = very true), the higher the score, the greater the athletes’ BPN. The Cronbach’s α coefficient of the scale was >0.85, and the Cronbach’s *α* coefficient of the scale was 0.831 in this study.

#### 2.2.4. Fear of failure

Fear of failure (FF) was measured using the Performance Failure Appraisal Inventory (PFAI; Conroy et al., 2002). This is a multidimensional measure of cognitive-motivational-relational appraisals associated with fear of failure. This measure consists of 25 items measuring beliefs associated with aversive consequences of failure. The stem for PFAI was related to performances in both sport and school. The PFAI has five subscales capturing: fear of experiencing shame and embarrassment (seven items; e.g., “When I am failing, it is embarrassing if others are there to see it”), fear of devaluing one’s self-estimate (four items; e.g., “When I am failing, I blame my lack of talent”), fear of important others losing interest (five items; e.g., “When I am not succeeding, people are less interested in me”), fear of upsetting important others (five items; “When I am failing, people who are important to me are disappointed”), and fear of having an uncertain future (four items; e.g., “When I am failing, it upsets my ‘plan’ for the future”). Participants provided answers using a five-point scale (0 = do not believe at all, 4 = believe 100% of the time). Mean scores were computed for each subscale of the PFAI, the higher the score, the greater the athletes’ FF. The Cronbach’s α coefficients of the scale’s five dimensions were 0.74–0.81, and the Cronbach’s α coefficient of the scale was 0.942 in this study.

### 2.3. Statistical analysis

Before analyzing the data, we performed a test for common method bias. The results of our Harman single-factor analysis showed that 12 factors with characteristic roots >1 were extracted from the unrotated exploratory factor analysis, and the maximum factor variance explanation rate was 25.872%, which was lower than the critical standard of 40% (Podsakoff et al., 2003). Therefore, there was no common methodological bias in this study.

The formal analyzes of the relationships were conducted in three steps. First, descriptive statistics, independent-sample *t*-test, one-way ANOVA, and Pearson’s correlation analysis between CCS, BPN, EC, CC, and FF were calculated in SPSS 26.0. Then, the measurement model was verified by conducting a confirmatory factor analysis (CFA) with maximum likelihood estimations. Three main judgment indicators were used: factor loading, combined reliability (CR), and average variance extraction (AVE). Finally, with maximum likelihood estimations, the latent variable structural equation modeling was used to determine the degree to which BPN, EC, and CC mediated the relationship between CCS and FF. The significance level of all variables was set as *α* = 0.05. Structural equation modeling analysis was completed with a 5,000 replication bootstrap with a 95% confidence interval. Data obtained in this study were analyzed using SPSS 26.0 and Mplus 8.0 software packages.

## 3. Results

### 3.1. Descriptive statistics

The comparison of differences in the various variables in this study is reported in Table 2. (1) There were significant differences in BPN and CC by gender, and CCS (*t* = 1.210, *p* > 0.05), EC (*t* = 1.721, *p* > 0.05), and FF (*t* = −1.403, *p* > 0.05) seemed to be unaffected by gender. It indicated that the scores of the males in BPN (*t* = 3.240, *p* < 0.01) were significantly higher than those of the females, while the females were significantly higher in CC (*t* = −2.249, *p* < 0.05) than the males; (2) CCS (*F* = 1.655, *p* > 0.05), BPN (*F* = 0.610, *p* > 0.05), EC (*F* = 2.357, *p* > 0.05), CC (*F* = 1.133, *p* > 0.05), and FF (*F* = 2.029, *p* > 0.05) seemed to be unaffected by age; (3) there were significant differences in CCS (*F* = 4.333, *p* < 0.05) and EC (*F* = 8.563, *p* < 0.01) by sports level, while BPN (*F* = 1.027, *p* > 0.05), CC (*F* = 1.903, *p* > 0.05), FF (*F* = 0.377, *p* > 0.05) had no significant differences by sports level. It indicated that the scores of elite athletes in CCS were significantly higher than those of national level 2 athletes, while the scores of national level 1 athletes and national level 2 athletes were significantly higher in EC than those of elite athletes; and (4) CCS (*t* = −1.2491, *p* > 0.05), BPN (*t* = 1.567, *p* > 0.05), EC (*t* = 1.767, *p* > 0.05), CC (*t* = −1.817, *p* > 0.05), and FF (*t* = −1.626, *p* > 0.05) seemed to be unaffected by sports events.

**Table 2:** Comparison of differences in the various variables (*n* = 252).

		CCS	BPN	EC	CC	FF
Gender	Male	3.290 ± 1.254	5.008 ± 0.839	4.320 ± 0.698	2.511 ± 0.970	3.560 ± 1.182
	Female	3.121 ± 0.952	4.652 ± 0.900	4.171 ± 0.677	2.788 ± 0.983	3.749 ± 0.950
	*t*-value	1.210	3.240^**^	1.721	−2.249^*^	−1.403
Age (years)	10–14	3.228 ± 1.001	4.747 ± 0.670	4.385 ± 0.578	2.944 ± 1.096	3.668 ± 1.092
	15–19	3.107 ± 1.053	4.808 ± 0.920	4.284 ± 0.650	2.632 ± 1.010	3.823 ± 0.990
	20–24	3.151 ± 1.137	4.788 ± 0.851	4.061 ± 0.711	2.565 ± 0.765	3.436 ± 0.969
	≥25	3.558 ± 1.267	4.997 ± 1.017	4.320 ± 0.807	2.642 ± 1.145	3.554 ± 1.373
	*F*-value	1.655	0.610	2.357	1.133	2.029
Sports level	Elite athlete①	3.517 ± 1.335	4.795 ± 0.949	3.960 ± 0.814	2.767 ± 0.869	3.570 ± 1.117
	Level 1②	3.178 ± 0.925	4.739 ± 0.839	4.294 ± 0.593	2.736 ± 0.950	3.721 ± 1.042
	Level 2③	3.007 ± 1.051	4.921 ± 0.886	4.390 ± 0.624	2.503 ± 1.077	3.663 ± 1.069
	*F*-value	4.333^*^	1.027	8.563^**^	1.903	0.377
	LSD	① > ③^**^	—	② > ①^**^	—	—
				③ > ①^***^		
Sports events	Group events	3.111 ± 1.124	4.915 ± 0.876	4.322 ± 0.731	2.537 ± 1.022	3.544 ± 1.187
	Individual events	3.285 ± 1.092	4.740 ± 0.893	4.169 ± 0.643	2.762 ± 0.941	3.762 ± 0.943
	*t*-value	−1.249	1.567	1.767	−1.817	−1.626

^*^p < 0.05, ^**^p < 0.01, ^***^p < 0.001; *t*-value, the value of the independent-sample t-test; *F*-value, the value of the one-way ANOVA test; LSD, least square difference. CCS, controlling coaching style; BPN, basic psychological need; CC, constrained commitment; EC, enthusiastic commitment; FF, fear of failure.

### 3.2. Correlation analysis

The mean, standard deviation, and correlation for the various variables in the study are reported in Table 3. CCS had a significantly positive correlation with CC (*r* = 0.364, *p* < 0.001) and FF (*r* = 0.345, *p* < 0.001), but a significantly negative correlation with BPN (*r* = −0.356, *p* < 0.001) and EC (*r* = −0.254, *p* < 0.001). BPN had a significantly positive correlation with EC (*r* = 0.249, *p* < 0.001), but a significantly negative correlation with CC (*r* = −0.377, *p* < 0.001) and FF (*r* = −0.493, *p* < 0.001). CC had a significantly positive correlation with FF (*r* = 0.420, *p* < 0.001), but a significantly negative correlation with EC (*r* = −0.157, *p* < 0.05). Lastly, there was a significantly negative correlation between EC and FF (*r* = −0.215, *p* < 0.01).

**Table 3:** Mean, standard deviation, and correlation matrix of the various variables (*n* = 252).

		*M* ± SD	1	2	3	4	5
1	CCS	3.202 ± 1.108	1				
2	BPN	4.823 ± 0.888	−0.356^***^	1			
3	CC	2.655 ± 0.985	0.364^***^	−0.377^***^	1		
4	EC	4.242 ± 0.690	−0.254^***^	0.249^***^	−0.157^*^	1	
5	FF	3.658 ± 1.070	0.345^***^	−0.493^***^	0.420^***^	−0.215^**^	1

^*^p < 0.05, ^**^p < 0.01, ^***^p < 0.001. *M*, mean; SD, standard deviation. CCS, controlling coaching style; BPN, basic psychological need; CC, constrained commitment; EC, enthusiastic commitment; FF, fear of failure.

### 3.3. Reliability and validity analysis

The reliability and convergent validity of the survey were tested before the questionnaire data are used for more in-depth analysis. Loading is used to ensure that the items provide an appropriate explanation for the factors. Combined reliability (CR) has the same meaning as Cronbach’s α and is examined to measure the internal consistency of the survey. A higher CR means that the survey has a better internal consistency. Average variance extracted (AVE) is necessary to check discriminant validity; thus, the AVE square root must be provided. According to the recommended standard of Chin (1998), Hair et al. (2021), and Fornell and Larcker (1981), most of the loadings should be at least 0.60 and ideally at 0.70 or above, indicating that each measure accounts for 50% or more of the variance of the underlying latent variable (Fornell and Larcker, 1981; Chin, 1998; Hair et al., 2021). For CR, 0.7 is an acceptable threshold. AVE should be greater than 0.5, but 0.36–0.5 is also acceptable. Additionally, the AVE square root must be greater than the correlations between the constructs.

As shown in Tables 4, 5, in Table 4, the standardized factor loadings for all structural variables are higher than 0.6 except ccs1, and significant at the *α* = 0.001, indicating that the scale has adequate convergent validity. CR of all structural variables is above the recommended level of 0.70 and AVE is also above the recommended level of 0.50, which indicates that this study has good reliability for the measurement of the structural variables in the study. The ccs1 was retained in this study because the CR and AVE indicators of the CCS scale were met, and the scale has been widely used (Ntoumanis et al., 2017) (e.g., Bartholomew et al., 2010; Ntoumanis et al., 2017; O’Neil and Hodge, 2020). In Table 5, the diagonal elements are greater than the off-diagonal elements in the corresponding rows and columns, indicating that each latent variable has adequate discriminant validity.

**Table 4:** Convergence validity.

Latent variable	Item	Unstd.	S.E.	*t*	*p*	Std.	SMC	CR	AVE	Cronbach’s alpha
CCS	ccs4	1				0.713	0.508	0.802	0.526	0.913
	ccs3	1.455	0.117	12.473	***	0.940	0.884			
	ccs2	1.186	0.103	11.546	***	0.765	0.585			
	ccs1	0.492	0.091	5.390	***	0.356	0.127			
BPN	bpn1	1				0.870	0.757	0.858	0.671	0.831
	bpn2	0.659	0.054	12.111	***	0.699	0.489			
	bpn3	0.966	0.063	15.353	***	0.875	0.766			
FF	ff1	1				0.837	0.701	0.863	0.559	0.942
	ff2	1.007	0.081	12.406	***	0.732	0.536			
	ff3	0.915	0.07	13.027	***	0.761	0.579			
	ff4	0.947	0.082	11.486	***	0.688	0.473			
	ff5	0.968	0.081	11.974	***	0.711	0.506			

^***^p < 0.001. Unstd., unstandardized coefficient; S.E., standard error of the covariance; Std, standardized coefficient; SMC, squared multiple correlation; CR, composite reliability; AVE, average variance extracted; CCS, controlling coaching style; BPN, basic psychological need; CC, constrained commitment; EC, enthusiastic commitment; FF, fear of failure.

**Table 5:** Discriminant validity.

	AVE	FF	BPN	CCS
FF	0.559	0.748		
BPN	0.671	−0.538	0.819	
CCS	0.526	0.387	−0.455	0.725

The diagonal data of the matrix represent the square root of the AVE values and the lower half of the matrix represents the correlation coefficient.

### 3.4. Model fit analysis

Goodness of fit (GOF) refers to the similarity of a theoretical model to the observed sample. The better the GOF, the closer the model matrix is to the sample matrix. To test the fit of the models, chi-squared by degrees of freedom ratio (χ^2/df^), goodness of fit index (GFI), and root mean square error of approximation (RMSEA) were used as absolute fit indexes. Incremental or comparative fit indexes were also considered by including the Tucker Lewis index (TLI) and the comparative fit index (CFI). Model fit is suggested to be acceptable when GFI ≥ 0.90, TLI ≥ 0.90, CFI ≥ 0.90, RMESA ≤0.08, and 1 < χ^2/df^ < 3 (Little, 2013).

The measurement model also exhibits an adequate model fit (χ^2/df^ = 1.890, RMSEA = 0.06, GFI = 0.927, TLI = 0.964, and CFI = 0.964). In sum, our test results indicate the appropriateness of the measurement model, which is a reliable indicator of the hypothesized constructs, thus allowing tests of the structural relationships in the various models to proceed.

### 3.5. Direct effects of independent variables

**Figure 2:**
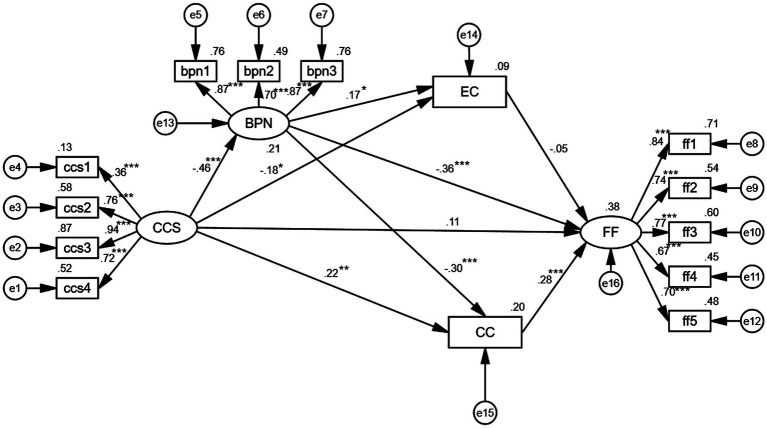
Path coefficient plot for the model. CCS, controlling coaching style; BPN, basic psychological need; CC, constrained commitment; EC, enthusiastic commitment; FF, fear of failure.

Because the GOF of the model is satisfactory, hypothesis testing is performed using Mplus 8.0 for path analysis to test the significance and coefficients of each path. As indicated by (Hair et al., 2021), the value of *p* must be below 0.05 and the critical ratio (C.R.) should be greater than 1.96 (Hair et al., 2021). According to Chin (1998), standardized path coefficients should be at least 0.20 and ideally above 0.30 to be meaningful. As shown in Table 6; Figure 2. The CCS had a significant impact on BPN, EC, and CC, with BPN (*γ* = −0.46, *p* < 0.001), EC (*γ* = −0.18, *p* < 0.05), and CC (*γ* = 0.22, *p* < 0.01), but had no significant impact on FF (*γ* = 0.11, *p* > 0.05). The BPN had a significant impact EC and CC, with EC (*β* = 0.17, p < 0.05) and CC (*β* = −0.30 *p* < 0.001). BPN and SC had a partial effect on FF, with BPN (*β* = −0.36, *p* < 0.001), CC (*β* = 0.28, *p* < 0.001) having a significant impact on FF, but EC (*β* = −0.05, *p* > 0.05) doing not have a significant impact. Thus, there is no significant influence relationship between EC and FF, and the relationship between BPN and EC and CCS and EC may be meaningless.

**Table 6:** Direct effects of independent variables.

Path	Std.	Unstd.	S.E.	C.R.	*p*
BPN ← CCS	−0.455	−0.441	0.070	−6.31	***
EC ← BPN	0.171	0.124	0.054	2.291	0.022
CC ← CCS	0.216	0.217	0.071	3.052	0.002
EC ← CCS	−0.183	−0.129	0.052	−2.469	0.014
CC ← BPN	−0.302	−0.314	0.074	−4.237	***
FF ← EC	−0.048	−0.071	0.088	−0.809	0.418
FF ← CC	0.279	0.292	0.067	4.352	***
FF ← BPN	−0.363	−0.394	0.082	−4.789	***
FF ← CCS	0.107	0.112	0.075	1.507	0.132

^***^p < 0.001. Std., standardized coefficient; Unstd., unstandardized coefficient; S.E., standard error of the covariance; C.R., critical ratio; CCS, controlling coaching style; BPN, basic psychological need; CC, constrained commitment; EC, enthusiastic commitment; FF, fear of failure.

### 3.6. Mediating effects analysis

The Bootstrap method currently serves as the ideal test method for mediating effects, if the upper and lower bounds do not include zero values in the 95% confidence interval, it indicates that there exists significant mediating effect (Preacher and Hayes, 2008). According to the results (Table 7), the values of the total indirect effect and total effect were 0.563 [CI (0.395, 0.899)] and 0.791 [CI (0.519, 1.260)], respectively, meaning that the total indirect effect accounted for 71.18% of the total effect. The mediating effect of BPN was significant; the value of the effect was 0.351 [CI (0.216, 0.655)] and the indirect effect accounted for 44.37% of the total effect. The mediating effect of EC was not significant [CI (−0.014, 0.106)]. The mediating effect of CC was significant; the value of the effect was 0.128 [CI (0.051, 0.261)] and the indirect effect accounted for 16.18% of the total effect. The chain mediating effect of BPN on EC was not significant [CI (−0.006, 0.052)]. However, the chain mediating effect of BPN on CC was significant; the value of the effect was 0.082 [CI (0.039, 0.168)] and the indirect effect accounted for 10.36% of the total effect. Additionally, research hypotheses 1–3 are supported.

**Table 7:** Bootstrap test results for the various paths of the model.

Predicted path	Estimate	SE	95% confidence interval	Proportion of total effect (%)
CCS → BPN → FF	0.351	0.126	[0.216, 0.655]	44.37
CCS → EC → FF	0.019	0.033	[−0.014, 0.106]	
CCS → CC → FF	0.128	0.063	[0.051, 0.261]	16.18
CCS → BPN → EC → FF	0.008	0.016	[−0.006, 0.052]	
CCS → BPN → CC → FF	0.082	0.037	[0.039, 0.168]	10.36
Total indirect effect	0.563	0.151	[0.395, 0.899]	71.18
Direct effect	0.227	0.177	[−0.024, 0.532]	
Total effect	0.791	0.226	[0.519, 1.260]	

SE, standard error; CCS, controlling coaching style; BPN, basic psychological need; CC, constrained commitment; EC, enthusiastic commitment; FF, fear of failure.

## 4. Discussion

Few studies have examined the role that gender plays in need satisfaction, and even fewer studies have examined gender differences in need satisfaction among high-level professional athletes (Nieves, 2021). This study found that BPN differed by gender, with male athletes having higher BPN than female athletes. According to emotional attribution theory, when feeling a negative experience, males complain about the external environment and females tend to complain about themselves. That is, females attribute more negative experiences to self-deficits and inadequacies, and due to differences in emotional brain structure, females also have a greater susceptibility to negative emotions, which may be more likely to result in lower basic psychological needs (Yuan et al., 2010; Qin, 2018). In addition, this study found that CC differed by gender, with females having higher constrained commitment than males. Lauderdale et al. (2015) found that females had higher forms of extrinsic regulation, rewards, constraints, and fear of punishment (Lauderdale et al., 2015). This may impact females in a way that leads to a stronger perception of obligation and higher constrained commitment in female athletes. There were no differences in age for CCS, EC, FF, BPN, and CC, which is consistent with some of the existing studies, but this study covered multiple sports, and the prime age of athletes is differ in different sports (Li, 2021). Therefore, the psychological differences brought by age may be masked by the type of sport and the age differences in the variables may be more appropriate to be explored in the context of a single sport. CCS and EC differed by sport level, with elite athletes having higher perceptions of CCS than national level 2 athletes and elite athletes having lower EC than athletes of other levels. The perpetual theme of performance-oriented competitive sports is higher, faster, and stronger. No matter how much athletes improve in terms of ability and tactical literacy as demonstrated in training, they are ultimately required to be further validated and recognized through competition. In a collectivist country like China, the performance of athletes is not only closely related to their own future career development but is also a matter of collective and national honor. Athletes at the elite level tend to face higher demands, more expectations, and stricter management, resulting in a higher level of perceived controlling coaching (Li, 2021). Athletes at the elite level are more mature psychologically and in terms of skills. They are concerned with consolidating their position on the team and improving their skills, and pursuing a stable career and life. Athletes at other levels still require upward mobility, psychologically and in terms of skills, and they are concerned with getting more opportunities to compete, getting better results, and realizing their athletic dreams (Cai and Wu, 2016). This may lead to a lower level of enthusiastic commitment among athletes at the elite level than athletes of other levels.

CCS was negatively correlated with athletes’ BPN satisfaction, which confirmed Hypothesis 1. This aligns with the findings of previous research (Curran et al., 2014; Yang et al., 2020), except for Pulido et al. (2018) and Bartholomew et al. (2011). This may be because: (i) in this study, the athletes’ BPN was measured based on the specific interpersonal context of coaches, preventing any underestimation of the relationship between those two variables; (ii) Chinese coaches often have the dual identities of coaches and pseudo-parents. They have to provide technical guidance, make training arrangements for the athletes, and care for and guide them through their lives and careers (Ye et al., 2016). Adopting a controlling style may decrease athletes’ BPN satisfaction.

Additionally, the results indicated that a CCS indirectly affected the athletes’ FF through their BPN. According to the SDT, environmental factors can affect self-integration and self-organization through the satisfaction level of an individual’s BPN (Deci and Ryan, 2000). Coaches with a controlling style tend to be authoritarian and enforce strict control over athletes’ behaviors and even belittle their abilities and contributions. In such an atmosphere, athletes demonstrate poorer BPN satisfaction and psychological well-being. Simultaneously, studies have shown that athletes’ perceptions of failure are not determined by good or bad sports performance but by how their needs are met (Conroy et al., 2001). Therefore, a CCS could lead to FF by affecting the athletes’ BPN.

After testing for Hypothesis 2, CCS was found to affect athletes’ FF through CC rather than EC. Studies show that EC are not associated with athletes’ FF, but CC are strong predictors of FF, social constraints are not associated with athletes’ EC but are strong predictors of CC. Athletes have a stronger sense of obligation to persevere after having experienced greater expectations from others (Wilson et al., 2004). A CCS might make athletes believe their behaviors are regulated by external factors and that they are obligated to participate in the sport rather than because of a passion for it, causing them to feel they “must” rather than “want” to participate in the sport. Those who participate in sports out of “obligation” tend to report lower levels of enjoyment and pay higher costs for effort (Schmidt and Stein, 1991), exhibiting a greater FF. Notably, the mediating effect of BPN was found to be higher than that of SC. BPN also accounted for a larger proportion of the total effect, indicating that these comprised a crucial mediating variable for a CCS’s effect on athletes’ FF.

For Hypothesis 3, it was found that a CCS reduced the BPN of athletes and further affected their FF. This occurred because of their affected CC rather than EC. The more controlling their coaches were perceived to be, the lower athletes’ BPN and the stronger the CC, leading to a worse FF. When athletes’ BPN was reduced because of coaches’ perceived control, their attitudes to FF varied depending on their SC. Those with CC viewed participation in high-performance sports as an obligation, which exacerbated their FF. By contrast, those with EC considered this a desire and were more willing to enjoy the competition process while caring less about the investment cost. Therefore, they were less prone to FF due to a CCS.

These findings contribute to a deeper understanding of why and how a controlling coaching style influences athletes’ fear of failure. There is an old Chinese saying that “a benevolent general does not manage his team well.” This reflects the traditional Chinese concept that authoritative and controlling leadership can have benign gains in organizational management. However, modern society has gradually realized the development of multi-ethnic cultural integration, and authoritarian leadership born from Confucianism and legalistic hierarchical culture may have lagged the development of the times. Thus, the concept of overly strict hierarchical culture does not always yield good results (Bartholomew et al., 2018; González-Hernández et al., 2019). In sports training, coaches should reduce the use of highly authoritative and multi-prescriptive training instructions, technical and tactical outcome feedback, and reinforcement feedback; reduce the use of punitive coaching such as punishment and reprimand; and use more technical and tactical process feedback, open-ended questions, praise, and Silent attention (Li et al., 2018). In addition, facing the diverse needs of athletes’ leadership styles, matching of diverse leadership styles should be achieved. Athletes of different genders and sport levels should receive different management and care styles. Furthermore, athletes are developing physically and mentally, and their needs are constantly changing. Perhaps, in the education and management of athletes, being oriented to the needs of athletes may be fundamental to completely improve the leadership effectiveness of coaches. Training excellent coaches is what is required for future sports development. Coaches cannot solely rely on experience, but also need theoretical support. Moreover, they must apply localized theories to exhibit the relationship between experience and theory in the field of Chinese sports. Currently, the research on coaching behavior in China is still in the exploration stage, and more research will be required in the future.

### 4.1. Limitations

This study has some limitations. First, during the COVID-19 pandemic, some sports teams declined our survey request in order to ensure the health of their athletes. The number of high-level athletes in a single sport was difficult to reach because of the sample size required for structural equation modeling studies; therefore, our study involved a sample of athletes from multiple sports. However, this entailed some new problems: the sample size of some sports is too small. For example, in rhythmic gymnastics, data were only found for 12 high-level athletes, and such a small sample may not be representative for rhythmic gymnastics players. In addition, collecting data samples from multiple sports also poses the problem of heterogeneity. To remedy these problems, this study included these data as a sample of group/individual events in the study and tried to achieve a balance in terms of sports events (group/individual). However, an analysis only in terms of sports events (group/individual) is insufficient. Future research must consider the unique characteristics of each sport and reduce the potential impact of program heterogeneity. Second, since young high-level athletes have become a trend, there are now many minors among high-level athletes. This study takes active high-level professional athletes as the research participants, which also involves a certain number of minor-aged participants. Although consent was sought from minor athletes, parents, and coaches, the study followed the principles of anonymity and voluntariness, and the paper-and-pencil questionnaire was used to ensure the accuracy of minor athletes’ understanding of the questionnaire and to avoid the influence of social expectations as much as possible. Because of the special characteristics of minor participants, future studies will need to consider using survey methods that are more appropriate to the psychological characteristics of minors to protect the interests of minor athletes, ensure the quality of communication between minors and adult researchers, and reduce understanding bias. Finally, this was a cross-sectional study, which lacked longitudinal data. As such, it was impossible to accurately infer the causal relationship between the variables. Future studies must examine the relationships between a CCS, BPN, the two types of SCs, and FF for temporal variations.

## 5. Conclusion

This study revealed the following findings. First, a CCS can predict athletes’ FF through BPN and indirectly predict their FF through CC. The mediating effect of BPN was found to be higher than that of CC. Second, BPN had a chain mediating effect on CC, mediated by a CCS and athletes’ FF, whereas the chain mediating effect of BPN on EC was not significant. The impact of a controlling coaching style on athletes’ fear of failure *via* basic psychological needs is manifested through the strengthening of constrained commitment rather than the weakening of enthusiastic commitment. This study enriches the research on athletes’ FF, and these findings provide useful insights not only for athlete development but also for future research in the field. It contributes to a deeper understanding of why and how a controlling coaching style influences athletes’ fear of failure. Coaches should carefully consider whether they are overusing control, actively understand the possible influences of different coaching styles, and seek more scientific and effective ways to instruct their athletes.

## Data availability statement

The raw data supporting the conclusions of this article will be made available by the authors, without undue reservation.

## Ethics statement

Ethical review and approval was not required for the study on human participants in accordance with the local legislation and institutional requirements. Written informed consent to participate in this study was provided by the participants’ legal guardian/next of kin.

## Author contributions

QH designed the research, conducted data analyzes, and wrote up the manuscript. PL was PI that contacted sports teams and organized data collection. BJ reviewed the literature and revised the manuscript. BL collected the data and collaborated on the data analysis. All authors contributed to the article and approved the submitted version.

## Conflict of interest

The authors declare that the research was conducted in the absence of any commercial or financial relationships that could be construed as a potential conflict of interest.

## Publisher’s note

All claims expressed in this article are solely those of the authors and do not necessarily represent those of their affiliated organizations, or those of the publisher, the editors and the reviewers. Any product that may be evaluated in this article, or claim that may be made by its manufacturer, is not guaranteed or endorsed by the publisher.

## References

[ref1] Adie J. W. Duda J. L. Ntoumanis N. (2008). Autonomy support, basic need satisfaction and the optimal functioning of adult male and female sport participants: a test of basic needs theory. Motiv. Emot. 32, 189–199. doi: 10.1007/s11031-008-9095-z

[ref2] Alvarez M. S. Balaguer I. Castillo I. Duda J. L. (2009). Coach autonomy support and quality of sport engagement in young soccer players. Span. J. Psychol. 12, 138–148. doi: 10.1017/s1138741600001554, PMID: 19476227

[ref3] Amorose A. J. Anderson-Butcher D. (2007). Autonomy-supportive coaching and self-determined motivation in high school and college athletes: a test of self-determination theory. Psychol. Sport Exerc. 8, 654–670. doi: 10.1016/j.psychsport.2006.11.003

[ref4] Balaguer I. Castillo I. Duda J. L. (2008). Apoyo a la autonomía, satisfacción de las necesidades, motivación y bienestar en deportistas de competición: un análisis de la teoría de la autodeterminación. Rev. Psicol. Deporte 17, 123–139.

[ref01] Bartholomew K. J. Ntoumanis N. Mouratidis A. Katartzi E. Thogersen-Ntoumani C. Vlachopoulos S. (2018). Beware of your teaching style: A school-year long investigation of controlling teaching and student motivational experiences. Learn. Instr. 53, 50–63. doi: 10.1016/j.learninstruc.2017.07.006

[ref5] Bartholomew K. J. Ntoumanis N. Thogersen-Ntoumani C. (2010). The controlling interpersonal style in a coaching context: development and initial validation of a psychometric scale. J. Sport Exerc. Psychol. 32, 193–216. doi: 10.1123/jsep.32.2.193, PMID: 20479478

[ref02] Bartholomew K. Ntoumanis N. Thøgersen-Ntoumani C. (2011). Self-determination theory and the darker side of athletic experience: The role of interpersonal control and need thwarting. Sport Exerc. Psychol. Rev. 7, 23–27. doi: 10.1177/0146167211413125

[ref6] Bélanger J. J. Lafreniere M. K. Vallerand R. J. Kruglanski A. W. (2013). Driven by fear: the effect of success and failure information on passionate individuals' performance. J. Pers. Soc. Psychol. 104, 180–195. doi: 10.1037/a0029585, PMID: 22889073

[ref7] Blanchard C. M. Amiot C. E. Perreault S. Vallerand R. J. Provencher P. (2009). Cohesiveness, coach's interpersonal style and psychological needs: their effects on self-determination and athletes' subjective well-being. Psychol. Sport Exerc. 10, 545–551. doi: 10.1016/j.psychsport.2009.02.005

[ref8] Boiché J. C. Sarrazin P. G. (2009). Proximal and distal factors associated with dropout versus maintained participation in organized sport. J. Sports Sci. Med. 8, 9–16. PMCID:, PMID: 24150550 PMC3737782

[ref9] Cai D. W. Wu Y. G. (2016). Mediating effect of basic psychological needs between perceived autonomy support and efforts willingness-a comparative analysis of adult and adolescent volleyball players. J. Xian Phys. Educ. Univ. 33, 113–118. doi: 10.16063/j.cnki.issn1001-747x.2016.01.018

[ref10] Carpenter P. J. Scanlan T. K. (1998). Changes over time in the determinants of sport commitment. Pediatr. Exerc. Sci. 10, 356–365. doi: 10.1123/pes.10.4.356

[ref11] Carpenter P. J. Scanlan T. K. Simons J. P. Lobel M. (1993). A test of the sport commitment model using structural equation modeling. J. Sport Exerc. Psychol. 15, 119–133. doi: 10.1123/jsep.15.2.119

[ref12] Chin W. W. (1998). Commentary: Issues and Opinion on Structural Equation Modeling. New York: JSTOR.

[ref13] Chu T. L. Zhang T. (2019). The roles of coaches, peers, and parents in athletes' basic psychological needs: a mixed-studies review. Int. J. Sports Sci. Coach. 14, 569–588. doi: 10.1177/1747954119858458

[ref14] Conroy D. E. Coatsworth J. D. Kaye M. P. (2007). Consistency of fear of failure score meanings among 8-to 18-year-old female athletes. Educ. Psychol. Meas. 67, 300–310. doi: 10.1177/0013164406288174

[ref15] Conroy D. E. Poczwardowski A. Henschen K. P. (2001). Evaluative criteria and consequences associated with failure and success for elite athletes and performing artists. J. Appl. Sport Psychol. 13, 300–322. doi: 10.1080/104132001753144428

[ref04] Conroy D. E. Willow J. P. Metzler J. N. (2002). Multidimensional fear of failure measurement: The performance failure appraisal inventory. J. Appl. Sport Psychol. 14, 76–90. doi: 10.1080/10413200252907752

[ref16] Correia M. E. Rosado A. (2018). Fear of failure and anxiety in sport. Anál. Psicol. 36, 75–86. doi: 10.14417/ap.1193

[ref17] Curran T. Hill A. P. Hall H. K. Jowett G. E. (2014). Perceived coach behaviors and athletes' engagement and disaffection in youth sport: the mediating role of the psychological needs. Int. J. Sport Psychol. 45, 559–580. doi: 10.7352/Ijsp2014.45.559

[ref18] De Muynck G. J. Soenens B. Delrue J. Comoutos N. Vansteenkiste M. (2020). Strengthening the assessment of self-talk in sports through a multi-method approach. Scand. J. Med. Sci. Sports 30, 602–614. doi: 10.1111/sms.13609, PMID: 31811733

[ref19] Deci E. L. Connell J. P. Ryan R. M. (1989). Self-determination in a work organization. J. Appl. Psychol. 74, 580–590. doi: 10.1037/0021-9010.74.4.580

[ref20] Deci E. L. Ryan R. M. (2000). The "what" and "why" of goal pursuits: human needs and the self-determination of behavior. Psychol. Inq. 11, 227–268. doi: 10.1207/S15327965pli1104_01

[ref21] Deci E. L. Ryan R. M. (2014). “Autonomy and need satisfaction in close relationships: relationships motivation theory,” in Human Motivation and Interpersonal Relationships: Theory, Research, and Applications, ed. Weinstein N.. 53–73.

[ref22] Fornell C. Larcker D. F. (1981). Evaluating structural equation models with unobservable variables and measurement error. J. Mark. Res. 18, 39–50. doi: 10.2307/3151312

[ref23] Gagne M. Ryan R. M. Bargmann K. (2003). Autonomy support and need satisfaction in the motivation and well-being of gymnasts. J. Appl. Sport Psychol. 15, 372–390. doi: 10.1080/714044203

[ref24] González-Hernández J. Muñoz-Villena A. J. (2019). Perfectionism and sporting practice. Functional stress regulation in adolescence. Ann. Psychol. 35, 148–155. doi: 10.6018/analesps.35.1.326541

[ref25] Gustafsson H. Sagar S. S. Stenling A. (2017). Fear of failure, psychological stress, and burnout among adolescent athletes competing in high level sport. Scand. J. Med. Sci. Sports 27, 2091–2102. doi: 10.1111/sms.12797, PMID: 27882607

[ref26] Hair J. F. Jr. Hult G. T. M. Ringle C. M. Sarstedt M. Danks N. P. Ray S. (2021). Partial Least Squares Structural Equation Modeling (PLS-SEM) Using R: A Workbook. Berlin: Springer Nature.

[ref27] Hodge K. Lonsdale C. (2011). Prosocial and antisocial behavior in sport: the role of coaching style, autonomous vs. controlled motivation, and moral disengagement. J. Sport Exerc. Psychol. 33, 527–547. doi: 10.1123/jsep.33.4.527, PMID: 21808078

[ref28] Hollembeak J. Amorose A. J. (2005). Perceived coaching behaviors and college athletes' intrinsic motivation: a test of self-determination theory. J. Appl. Sport Psychol. 17, 20–36. doi: 10.1080/10413200590907540

[ref29] Horn T. (2002). Coaching effectiveness in sport domain: advances in sport psychology. J. Psychol. 7, 134–149.

[ref30] Jackson D. L. (2003). Revisiting sample size and number of parameter estimates: some support for the N: q hypothesis. Struct. Equ. Model. Multidiscip. J. 10, 128–141. doi: 10.1207/S15328007sem1001_6

[ref31] Keegan R. Spray C. Harwood C. Lavallee D. (2010). The motivational atmosphere in youth sport: coach, parent, and peer influences on motivation in specializing sport participants. J. Appl. Sport Psychol. 22, 87–105. doi: 10.1080/10413200903421267

[ref32] Keegan R. J. Harwood C. G. Spray C. M. Lavallee D. E. (2009). A qualitative investigation exploring the motivational climate in early career sports participants: coach, parent and peer influences on sport motivation. Psychol. Sport Exerc. 10, 361–372. doi: 10.1016/j.psychsport.2008.12.003

[ref03] La Guardia J. G. Ryan R. M. Couchman C. E. Deci E. L. (2000). Within-person variation in security of attachment: a self-determination theory perspective on attachment, need fulfillment, and well-being. J. Pers. Soc. Psychol. 79:367. doi: 10.1037/0022-3514.79.3.367 10981840

[ref33] Li J. (2021). *Research on the Influencing Mechanism of Paternalistic Leadership on Youth Football Players’ Behavior*. Doctoral Dissertation, Beijing Jiaotong University.

[ref34] Li Q. Han Y. Li W. Z. (2018). Research on training mode and coaching behaviors of youth soccer coaches. China Sport Sci. 38:10. doi: 10.16469/j.css.201802004

[ref35] Little T. D. (2013). Longitudinal structural equation modeling. New York: Guilford Press

[ref36] Lauderdale M. E. Yli-Piipari S. Irwin C. C. Layne T. E. (2015). Gender differences regarding motivation for physical activity among college students: a self-determination approach. Phys. Educ. 72, 153–172. doi: 10.18666/TPE-2015-V72-I5-4682

[ref37] Madigan D. J. Stoeber J. Passfield L. (2016). Motivation mediates the perfectionism-burnout relationship: a three-wave longitudinal study with junior athletes. J. Sport Exerc. Psychol. 38, 341–354. doi: 10.1123/jsep.2015-0238, PMID: 27383053

[ref38] Mageau G. A. Vallerand R. J. (2003). The coach-athlete relationship: a motivational model. J. Sports Sci. 21, 883–904. doi: 10.1080/0264041031000140374, PMID: 14626368

[ref39] Martin A. J. Marsh H. W. (2003). Fear of failure: friend or foe? Aust. Psychol. 38, 31–38. doi: 10.1080/00050060310001706997

[ref40] Nieves J. (2021). Understanding the Influence of Gender and Psychological Factors on College Students’ Exercise Behavior, California State University, Bakersfield.

[ref41] Ntoumanis N. Barkoukis V. Gucciardi D. F. Chan D. K. C. (2017). Linking coach interpersonal style with athlete doping intentions and doping use: a prospective study. J. Sport Exerc. Psychol. 39, 188–198. doi: 10.1123/jsep.2016-0243, PMID: 28891379

[ref42] Ng J. Y. Lonsdale C. Hodge K. (2011). The basic needs satisfaction in sport scale (BNSSS): instrument development and initial validity evidence. Psychol. Sport Exerc. 12, 257–264. doi: 10.1016/j.psychsport.2010.10.006 18648109

[ref43] O’Neil L. Hodge K. (2020). Commitment in sport: the role of coaching style and autonomous versus controlled motivation. J. Appl. Sport Psychol. 32, 607–617. doi: 10.1080/10413200.2019.1581302

[ref44] Olusoga P. Kentta G. (2017). Desperate to quit: a narrative analysis of burnout and recovery in high-performance sports coaching. Sport Psychol. 31, 237–248. doi: 10.1123/tsp.2016-0010

[ref45] Podsakoff P. M. MacKenzie S. B. Lee J.-Y. Podsakoff N. P. (2003). Common method biases in behavioral research: a critical review of the literature and recommended remedies. J. Appl. Psychol. 88, 879–903. doi: 10.1037/0021-9010.88.5.879, PMID: 14516251

[ref46] Preacher K. J. Hayes A. F. (2008). Asymptotic and resampling strategies for assessing and comparing indirect effects in multiple mediator models. Behav. Res. Methods 40, 879–891. doi: 10.3758/brm.40.3.879, PMID: 18697684

[ref47] Pulido J. J. Sanchez-Oliva D. Sanchez-Miguel P. A. Amado D. Garcia-Calvo T. (2018). Sport commitment in young soccer players: a self-determination perspective. Int. J. Sports Sci. Coach. 13, 243–252. doi: 10.1177/1747954118755443

[ref48] Qin S. S. (2018). An analysis of the intergenerational transmission of gender role concepts in China. Chin. J. Popul. Sci. 14:8.

[ref06] Raabe J. Zakrajsek R. A. (2017). Coaches and teammates as social agents for collegiate athletes’ basic psychological need satisfaction. J. Intercolleg. Sport. 10, 67–82. doi: 10.1123/jis.2016-0033

[ref49] Reinboth M. Duda J. L. (2006). Perceived motivational climate, need satisfaction and indices of well-being in team sports: a longitudinal perspective. Psychol. Sport Exerc. 7, 269–286. doi: 10.1016/j.psychsport.2005.06.002

[ref50] Ryan R. M. Deci E. L. (2000). Self-determination theory and the facilitation of intrinsic motivation, social development, and well-being. Am. Psychol. 55, 68–78. doi: 10.1037//0003-066x.55.1.68, PMID: 11392867

[ref51] Ryan R. M. Deci E. L. (2017). Self-Determination Theory: Basic Psychological Needs in Motivation, Development, and Wellness. New York: Guilford Publications.

[ref52] Sagar S. S. Boardley I. D. Kavussanu M. (2011). Fear of failure and student athletes' interpersonal antisocial behaviour in education and sport. Br. J. Educ. Psychol. 81, 391–408. doi: 10.1348/2044-8279.002001, PMID: 21199481

[ref53] Sagar S. S. Lavallee D. Spray C. M. (2007). Why young elite athletes fear failure: consequences of failure. J. Sports Sci. 25, 1171–1184. doi: 10.1080/02640410601040093, PMID: 17654229

[ref54] Sagar S. S. Lavallee D. Spray C. M. (2009). Coping with the effects of fear of failure: a preliminary investigation of young elite athletes. J. Clin. Sport Psychol. 3, 73–98. doi: 10.1123/jcsp.3.1.73

[ref55] Santi G. Bruton A. Pietrantoni L. Mellalieu S. (2014). Sport commitment and participation in masters swimmers: the influence of coach and teammates. Eur. J. Sport Sci. 14, 852–860. doi: 10.1080/17461391.2014.915990, PMID: 24826953

[ref56] Scanlan T. K. Chow G. M. Sousa C. Scanlan L. A. Knifsend C. A. (2016). The development of the sport commitment Questionnaire-2 (English version). Psychol. Sport Exerc. 22, 233–246. doi: 10.1016/j.psychsport.2015.08.002

[ref57] Scanlan T. K. Russell D. G. Beals K. P. Scanlan L. A. (2003). Project on elite athlete commitment (PEAK): II. A direct test and expansion of the sport commitment model with elite amateur sportsmen. J. Sport Exerc. Psychol. 25, 377–401. doi: 10.1123/jsep.25.3.377

[ref58] Scanlan T. K. Russell D. G. Scanlan L. A. Klunchoo T. J. Chow G. M. (2013). Project on elite athlete commitment (PEAK): IV. Identification of new candidate commitment sources in the sport commitment model. J. Sport Exerc. Psychol. 35, 525–535. doi: 10.1123/jsep.35.5.525, PMID: 24197720

[ref59] Schmidt G. W. Stein G. L. (1991). Sport commitment - a model integrating enjoyment, dropout, and burnout. J. Sport Exerc. Psychol. 13, 254–265. doi: 10.1123/jsep.13.3.254

[ref60] Si G. Duan Y. Li H. Y. Jiang X. (2011). An exploration into socio-cultural meridians of Chinese athletes’ psychological training. J. Clin. Sport Psychol. 5, 325–338. doi: 10.1123/jcsp.5.4.325

[ref61] Smith R. E. Smoll F. L. Schutz R. W. (1990). Measurement and correlates of sport-specific cognitive and somatic trait anxiety: the sport anxiety scale. Anxiety Res. 2, 263–280. doi: 10.1080/08917779008248733

[ref62] Sousa C. Torregrosa M. Viladrich C. Villamarin F. Cruz J. (2007). The commitment of young soccer players. Psicothema 19, 256–262. 17425896

[ref05] Vallerand R. J. Briere N. M. Blanchard C. Provencher P. (1997). Development and validation of the multidimensional sportspersonship orientations scale. J. Sport Exerc. Psychol. 19, 197–206. doi: 10.1123/jsep.19.2.197

[ref64] Wilson P. M. Rodgers W. M. Carpenter P. J. Hall C. Hardy J. Fraser S. N. (2004). The relationship between commitment and exercise behavior. Psychol. Sport Exerc. 5, 405–421. doi: 10.1016/S1469-0292(03)00035-9

[ref63] Yang W. Zhai F. Gao Y. (2020). Psychological toughness and basic psychological needs: factors influencing the emotional expression and athletic performance of athletes and mediating effect test. J. Xian Inst. Phys. Educ. 4, 488–496. doi: 10.16063/j.cnki.issn1001-747x.2020.04.016

[ref65] Ye L. Wang B. Liu Z. Wu Y. Dong L. (2016). The effect of coach-athlete relationship on sport performance satisfaction–serial multiple mediating effects of hope and athele engagement. China Sport Sci. 7, 40–48. doi: 10.16469/j.css.201607005

[ref66] Young B. W. Medic N. (2011). Examining social influences on the sport commitment of masters swimmers. Psychol. Sport Exerc. 12, 168–175. doi: 10.1016/j.psychsport.2010.09.004

[ref67] Yuan J. J. Wang Y. Ju E. X. Li H. (2010). Gender differences in emotional processing and its neural mechanisms advances. Psychol. Sci. 18, 1899–1908.

[ref68] Zhang B. Yu L. F. (2021). Bridging need-support dimensions to Athlete's intentions to continue sports through enthusiastic and constrained commitment: an empirical study in China. Rev. Psicol. Deporte 30, 152–164.

